# Construction and Validation of a Lung Cancer Risk Prediction Model for Non-Smokers in China

**DOI:** 10.3389/fonc.2021.766939

**Published:** 2022-01-04

**Authors:** Lan-Wei Guo, Zhang-Yan Lyu, Qing-Cheng Meng, Li-Yang Zheng, Qiong Chen, Yin Liu, Hui-Fang Xu, Rui-Hua Kang, Lu-Yao Zhang, Xiao-Qin Cao, Shu-Zheng Liu, Xi-Bin Sun, Jian-Gong Zhang, Shao-Kai Zhang

**Affiliations:** ^1^ Department of Cancer Epidemiology and Prevention, Henan Engineering Research Center of Cancer Prevention and Control, Henan International Joint Laboratory of Cancer Prevention, Henan Cancer Hospital, The Affiliated Cancer Hospital of Zhengzhou University, Zhengzhou, China; ^2^ Department of Cancer Epidemiology and Biostatistics, National Clinical Research Center for Cancer, Key Laboratory of Cancer Prevention and Therapy of Tianjin, Tianjin’s Clinical Research Center for Cancer, Key Laboratory of Molecular Cancer Epidemiology of Tianjin, Key Laboratory of Breast Cancer Prevention and Therapy of the Ministry of Education, Tianjin Medical University Cancer Institute and Hospital, Tianjin, China; ^3^ Department of Radiology, Henan Cancer Hospital, The Affiliated Cancer Hospital of Zhengzhou University, Zhengzhou, China

**Keywords:** lung cancer, risk model, forecasting, validation, non-smokers

## Abstract

**Background:**

About 15% of lung cancers in men and 53% in women are not attributable to smoking worldwide. The aim was to develop and validate a simple and non-invasive model which could assess and stratify lung cancer risk in non-smokers in China.

**Methods:**

A large-sample size, population-based study was conducted under the framework of the Cancer Screening Program in Urban China (CanSPUC). Data on the lung cancer screening in Henan province, China, from October 2013 to October 2019 were used and randomly divided into the training and validation sets. Related risk factors were identified through multivariable Cox regression analysis, followed by establishment of risk prediction nomogram. Discrimination [area under the curve (AUC)] and calibration were further performed to assess the validation of risk prediction nomogram in the training set, and then validated by the validation set.

**Results:**

A total of 214,764 eligible subjects were included, with a mean age of 55.19 years. Subjects were randomly divided into the training (107,382) and validation (107,382) sets. Elder age, being male, a low education level, family history of lung cancer, history of tuberculosis, and without a history of hyperlipidemia were the independent risk factors for lung cancer. Using these six variables, we plotted 1-year, 3-year, and 5-year lung cancer risk prediction nomogram. The AUC was 0.753, 0.752, and 0.755 for the 1-, 3- and 5-year lung cancer risk in the training set, respectively. In the validation set, the model showed a moderate predictive discrimination, with the AUC was 0.668, 0.678, and 0.685 for the 1-, 3- and 5-year lung cancer risk.

**Conclusions:**

We developed and validated a simple and non-invasive lung cancer risk model in non-smokers. This model can be applied to identify and triage patients at high risk for developing lung cancers in non-smokers.

## Highlights

1. Evidence before this study:
• About 15% of lung cancers in men and 53% in women are not attributable to smoking worldwide.
• Screening people at high risk for lung cancer by low-dose computed tomography (LDCT) has been approved to effective in reducing the burden of this disease.
• Developing lung-cancer risk prediction tools for Chinese non-smokers in large-scale population-based lung screening programs is sparse.
2. Added value of this study:
• Risk factors associated with lung cancer in Chinese non-smokers were identified.
• The model developed has moderate discriminatory accuracy and goodness-of-fit for both men and women, non-passive smokers and passive smokers.
3. Implications of all the available evidence:
• This model can be applied to identify and triage patients at high risk for developing lung cancer in non-smokers.
• The model has potential utility for shared decision making and individualized risk assessment for tailored lung cancer screening in Chinese non-smokers.

## Introduction

Lung cancer is the leading cause of cancer related deaths in both the world and China. The latest data from the International Agency for Research on Cancer (IARC) shows that in 2020, there were about 1.80 million lung cancer deaths worldwide, which China accounts for 39.8% (1). The majority of lung cancer cases in China were found to be clinically advanced, with 64.6% of stage III-IV lung cancers in 2012-2014 (2). The age standardized 5-year survival rate of lung cancer in China increased slightly between 2003 and 2015, but still did not exceed 20.0% (3). The prognosis of lung cancer is closely related to the diagnostic stage, and the 5-year survival rate after surgery is almost 0 for stage IV patients, but >80% for stage I lung cancer patients (4).

The results of the National Lung Screening Trial (NLST), initiated in 2002, suggested that low-dose computed tomography (LDCT) screening could reduce lung cancer mortality by 20% (5). However, this project only screened people at high risk for lung cancer based on age and smoking history (55-74 years, smoked no less than 30 pack-years, and had no more than 15 years of smoking quit time). It is well known that smoking significantly increases the risk of lung cancer. Meta-analysis showed that the risk of lung cancer was 13.1 times higher among smokers than non-smokers in Europe and the United States [Hazard Ratio (HR)=13.1, 95% CI= 9.9-17.3] (6), much higher than the 2.77 times risk in the Chinese population [Odds Ratio (OR)=2.77, 95% CI=2.26-3.40] (7). This suggests that the current international standards for lung cancer screening based on smoking as the main indicator for high-risk populations may not be suitable for the Chinese population, especially for Chinese non-smokers. Therefore, how to effectively predict the risk of lung cancer in non-smokers and then guide the more cost-effective LDCT screening is an effective way to achieve efficient early diagnosis and treatment of lung cancer.

Previous studies have constructed several lung cancer risk prediction models based on different characteristics of populations (8–38), but there is few lung cancer risk prediction models based on non-smokers in mainland of China. To this end, developing lung-cancer risk prediction tools for Chinese non-smokers based on risk factors consistently identified in previous studies becomes a priority (39). However, this is difficult and challenging. Unlike the situation of tobacco-driven lung cancer, there is no established risk factors dominating the development of lung cancer among non-smokers. Numerous risk factors have been suggested and their effects vary greatly by geographical region (40–43). For example, we note that the Prostate, Lung, Colorectal, and Ovarian Cancer Screening Trial (PLCO) models do not seem to be useful for Asian non-smokers because PLCO only included about 2000 never-smokers of Asian ethnicity, of which 7 cases of lung cancer occurred (44). Indeed, none of the non-smokers in the PLCO (n=65,711) had a six-year risk >0.0151, using the PLCO_M2014_ that is analogous to PLCO_M2012_ and included non-smokers.

The model was developed based on the Cancer Screening Program in Urban China (CanSPUC) (45). With the focus on established risk factors for lung cancer routinely available in general cancer screening settings, we aimed to develop and internally validated a risk prediction model for lung cancer in Chinese non-smokers.

## Methods

### Data Source and Subjects

This study was conducted within the framework of CanSPUC, an ongoing, nationwide, population-based cancer screening program in urban China. The purpose of CanSPUC is to screen five most prevalent cancers, including lung cancer, female breast cancer, liver cancer, upper gastrointestinal cancer, and colorectal cancer. The methodology of the CanSPUC has been previously described (45, 46). In brief, after signing a written informed consent, all eligible participants (40-74 years old) were interviewed by trained staffs to collect data on their exposure to risk factors and to evaluate their cancer risk using a defined clinical cancer risk score system. CanSPUC was launched in Henan province of China in October 2013, covering eight cities (Zhengzhou, Zhumadian, Anyang, Luoyang, Nanyang, Jiaozuo, Puyang, and Xinxiang). In this study, we used data from the first six years (from October 2013 to October 2019) in Henan province. Only those non-smokers (except former smokers) were included in this study. Subjects would be excluded if they have been already diagnosed with lung cancer.

### Outcome, Variables and Measurements

All new cases of lung cancer in the study were ascertained through local cancer registry databases with a histologically confirmed diagnosis from October 1, 2013 to March 10, 2020 in mainland of China. Newly diagnosed lung cancers were classified by sites according to International Classification of Diseases, 10th version (ICD-10). Lung cancers were identified by ICD-10 of C33-C34. To identify potential risk factors for lung cancer, the following data were collected by self-report:

(1) Demographic characteristics: including age, gender, race, height, weight and level of education. A low education was defined as primary school or below, medium education was defined junior or senior high school, and high-level education was defined as undergraduate or over. Body mass index (BMI) was calculated according to height and weight, and classified as “<18.5 kg/m^2^”, “18.5-23.9 kg/m^2^”, “24.0-27.9 kg/m^2^”and “≥28.0 kg/m^2^”.
(2) Dietary habit: a) Dietary intake of the following food in the past two years: vegetables intake (<2.5kg/week, ≥2.5kg/week), fruit intake (<1.25kg/week, ≥1.25kg/week), roughage intake (<0.5kg/week, ≥0.5kg/week). Vegetables referred to green leaf plants and fungi, except for potato, sweet potato, and other starch. Roughage referred to the grains except white flour and rice. Food weight was determined before cooking. b) Taste preferences: heavy-salt diet (yes, no) and heavy-grease diet (yes, no).
(3) Living environment, behavior and habits: a) Cooking oil fume (COF) exposure: exposure is considered as “none or a little”, if chimneys, fume extractors, or smoke-less pots was used during cooking; otherwise, it is considered as “a lot”. b) Physical activity: activities were categorized as Taijiquan/Qigong/Walking, long distance running/aerobics, ball games (basketball, table tennis, badminton, etc.), fast walking/yangko dance, swimming and other physical exercises (such as mountain climbing, rope skipping, shuttlecock kicking). Subjects who did exercise for at least three days with a total time ≥90 mins per week were categorized as “heavy physical activity”; otherwise, were categorized as “moderate or no physical activity”.
(4) Comorbidities: including history of chronic respiratory disease, tuberculosis, chronic bronchitis, emphysema, asthma bronchiectasis and hyperlipidemia. All self-reported comorbidities required a diagnosis from professional medical institutions.
(5) Family history of lung cancer: whether first-degree relatives, second-degree relatives or third-degree relatives had lung cancer or not.

### Statistical Analysis

All statistical analyses were performed with the statistical software SAS version 9.4 (SAS Institute, Cary, NC) and R version 4.0.3 (The Free Software Foundation, Boston, MA, USA). The “rms” package was used to draw the nomogram. The “survivalROC” package was used to draw the ROC curves. The “ggplot2” package was used to draw the calibration curves. All tests were two-sided and p-values of 0.05 or less were considered to be statistically significant.

With the help of randomization codes produced by means of the PROC PLAN of the SAS system, the dataset was randomly divided into training set and validation set with a 1:1 assignment ratio. The training set was used to create the risk prediction model, while the validation set was used to validate the performance of the model.

Descriptive statistics, expressed as proportions for categorical variables, were used to compare the characteristics of those with and without the outcome of developing lung cancer. Chi-squared tests for categorical variables were used to determine the univariate association between the baseline factors and lung cancer development. Continuous variables were described by means (standard deviation) or median (interquartile range, IQR).

In this study, the combined model based on all independent prognostic factors selected by the stepwise multivariable Cox regression (*P*_entry_=0.15, *P*_stay_=0.10) was used to construct a nomogram to assess the 1‐, 3‐, and 5‐year estimates of the lung cancer risk in the training set. The calibration curves were used to evaluate the validity of the nomogram. The Kaplan–Meier curves were plotted for low-, medium-, and high-risk groups using the 33% and 66% quantiles for lung cancer according to the risk prediction model, and differences among the three curves were tested according to the log-rank test. The prediction performance of the 1-, 3- and 5-year estimates of the lung cancer risk was quantified by receiver operating characteristic (ROC) curves and the area under the curve (AUC) in the training set and validation set. The bootstrap sampling approach was used to evaluate the calibration of the present model by comparing the observed and predicted probabilities. Correction for deviation of estimates from observations (overfitting correction) estimates were based on predictions for a subset of the interval. The median absolute error is also used to evaluate the calibration performance.

## Results

### Characteristics of the Study Population

**Figure 1:**
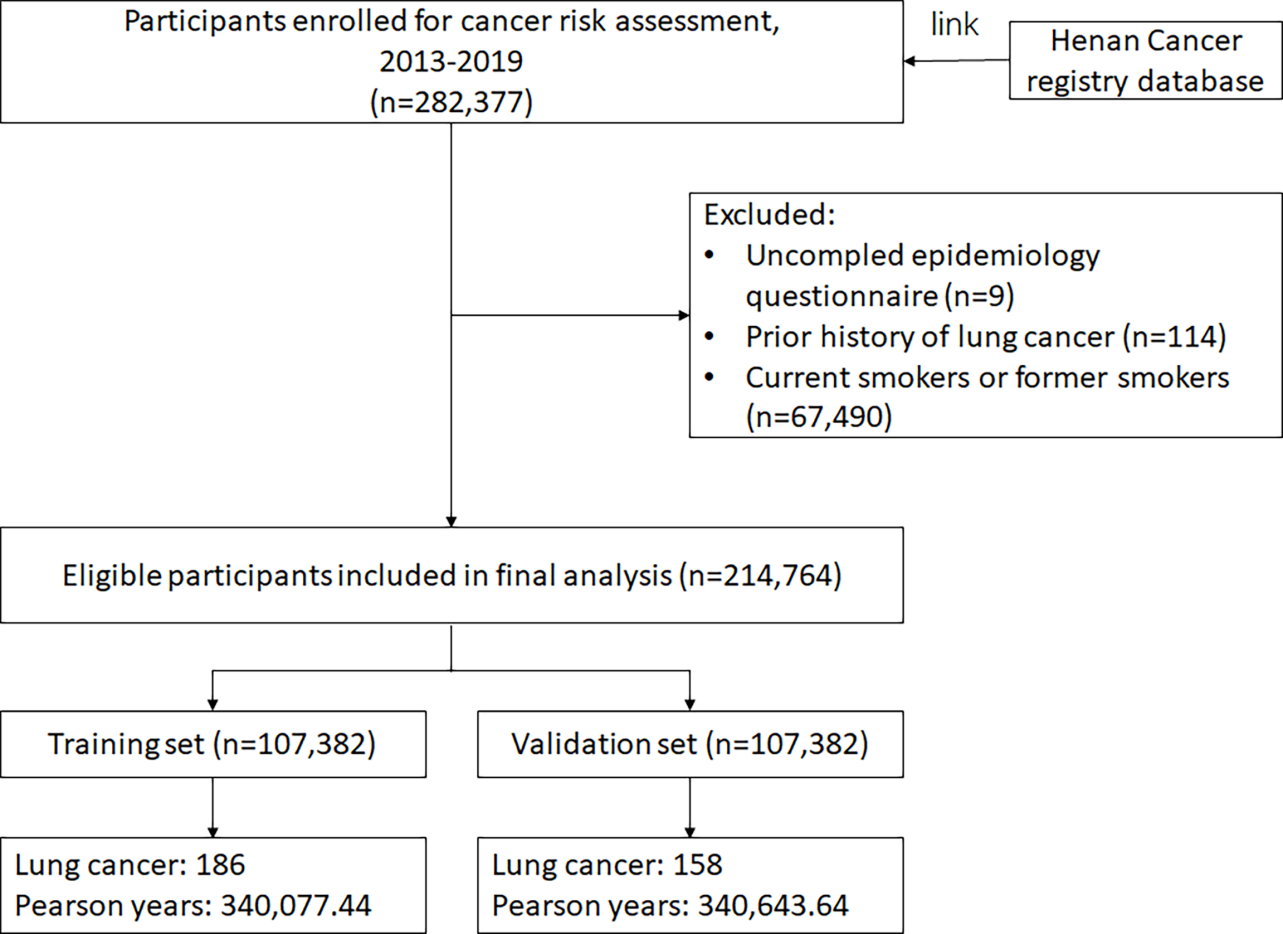
Flow chart of participants included in this analysis.

A total of 214,764 eligible subjects with a mean age of 55.19 years were included into this study, and 70.70% were females. Subjects were randomly divided into the training set (107,382 subjects) and validation set (107,382 subjects) (**Figure 1**). By March 2020, among 214,764 eligible participants, 344 lung cancer cases occurred in the follow-up yielding an incident density of 50.53/100,000 person-years. Compared with participants without lung cancer, lung cancer cases were more likely to have a low education, without passive smoking exposure, have a heavy physical activity and have a family history of lung cancer (all *P* vales <0.05). Additional characteristics are presented in **Supplementary Table 1** and **Table 1**.

**Table 1:** Baseline characteristics of the study population in the training set.

	Total no. (%)	No lung cancer, n (%)	Lung cancer, n (%)	χ^2^	*P-*value
All participants	107382	107196	186		
Person-years, median(IQR)	2.95 (1.71-4.83)	2.95 (1.71-4.83)	1.47 (0.78-2.33)		
Age, mean ± SD, years	55.16 ± 8.78	55.14 ± 8.78	61.53 ± 7.47		
Age (years)				98.24	<0.001
40-44	14028 (13.06)	14021 (99.95)	7 (0.05)		
45-49	20109 (18.73)	20101 (99.96)	8 (0.04)		
50-54	19769 (18.41)	19750 (99.90)	19 (0.10)		
55-59	16083 (14.98)	16054 (99.82)	29 (0.18)		
60-64	17556 (16.35)	17508 (99.73)	48 (0.27)		
65-69	14356 (13.37)	14302 (99.62)	54 (0.38)		
70-74	5481 (5.10)	5460 (99.62)	21 (0.38)		
Gender				16.73	<0.001
Male	31531 (29.36)	31451 (99.75)	80 (0.25)		
Female	75851 (70.64)	75745 (99.86)	106 (0.14)		
Race				0.44	0.506
Han nationality	105549 (98.29)	105365 (99.83)	184 (0.17)		
Others	1833 (1.71)	1831 (99.89)	2 (0.11)		
Education^a^				14.34	0.001
Low	20139 (18.75)	20086 (99.74)	53 (0.26)		
Medium	71634 (66.71)	71517 (99.84)	117 (0.16)		
High	15609 (14.54)	15593 (99.90)	16 (0.10)		
BMI (kg/m^2^)				2.82	0.419
<18.5	1381 (1.29)	1377 (99.71)	4 (0.29)		
18.5-23.9	47588 (44.32)	47498 (99.81)	90 (0.19)		
24.0-28.0	46882 (43.66)	46810 (99.85)	72 (0.15)		
≥28.0	11531 (10.74)	11511 (99.83)	20 (0.17)		
Vegetables intake				0.03	0.861
≥2.5kg/week	56467 (52.59)	56368 (99.82)	99 (0.18)		
<2.5kg/week	50915 (47.41)	50828 (99.83)	87 (0.17)		
Fruit intake				0.07	0.785
≥1.25kg/week	63026 (58.69)	62915 (99.82)	111 (0.18)		
<1.25kg/week	44356 (41.31)	44281 (99.83)	75 (0.17)		
Roughage intake				0.47	0.492
≥0.5kg/week	73524 (68.47)	73401 (99.83)	123 (0.17)		
<0.5kg/week	33858 (31.53)	33795 (99.81)	63 (0.19)		
Heavy-slat diet				1.57	0.209
No	88623 (82.53)	88463 (99.82)	160 (0.18)		
Yes	18759 (17.47)	18733 (99.86)	26 (0.14)		
Heavy-grease diet				0.07	0.793
No	89672 (83.51)	89518 (99.83)	154 (0.17)		
Yes	17710 (16.49)	17678 (99.82)	32 (0.18)		
Cooking oil fume exposure				0.03	0.853
None or a little	95144 (88.60)	94980 (99.83)	164 (0.17)		
A lot	12238 (11.40)	12216 (99.82)	22 (0.18)		
Passive smoking				4.65	0.031
No	76618 (71.35)	76472 (99.81)	146 (0.19)		
Yes	30764 (28.65)	30724 (99.87)	40 (0.13)		
Alcohol Drinking				0.46	0.794
Never	96004 (89.40)	95835 (99.82)	169 (0.18)		
Current	9598 (8.94)	9584 (99.85)	14 (0.15)		
Former	1780 (1.66)	1777 (99.83)	3 (0.17)		
Physical activity				3.38	0.066
Moderate or no	56293 (52.42)	56208 (99.85)	85 (0.15)		
Heavy	51089 (47.58)	50988 (99.80)	101 (0.20)		
Family history of lung cancer				4.69	0.030
No	99660 (92.81)	99495 (99.83)	165 (0.17)		
Yes	7722 (7.19)	7701 (99.73)	21 (0.27)		
History of chronic respiratory disease				1.93	0.165
No	93185 (86.78)	93030 (99.83)	155 (0.17)		
Yes	14197 (13.22)	14166 (99.78)	31 (0.22)		
History of tuberculosis				4.58	0.032
No	106230 (98.93)	106049 (99.83)	181 (0.17)		
Yes	1152 (1.07)	1147 (99.57)	5 (0.43)		
History of chronic bronchitis				1.55	0.213
No	96485 (89.85)	96323 (99.83)	162 (0.17)		
Yes	10897 (10.15)	10873 (99.78)	24 (0.22)		
History of emphysema				4.51	0.034
No	106543 (99.22)	106361 (99.83)	182 (0.17)		
Yes	839 (0.78)	835 (99.52)	4 (0.48)		
History of asthma bronchiectasis				0.01	0.925
No	104612 (97.42)	104431 (99.83)	181 (0.17)		
Yes	2770 (2.58)	2765 (99.82)	5 (0.18)		
History of hyperlipidemia				0.50	0.481
No	91561 (85.27)	91399 (99.82)	162 (0.18)		
Yes	15821 (14.73)	15797 (99.85)	24 (0.15)		

^a^Low, primary school or below; Medium, junior or senior high school; High, undergraduate or over.

IQR, Interquartile range; BMI, body mass index.

### Development of the Lung Cancer Risk Assessment Model

**Figure 2:**
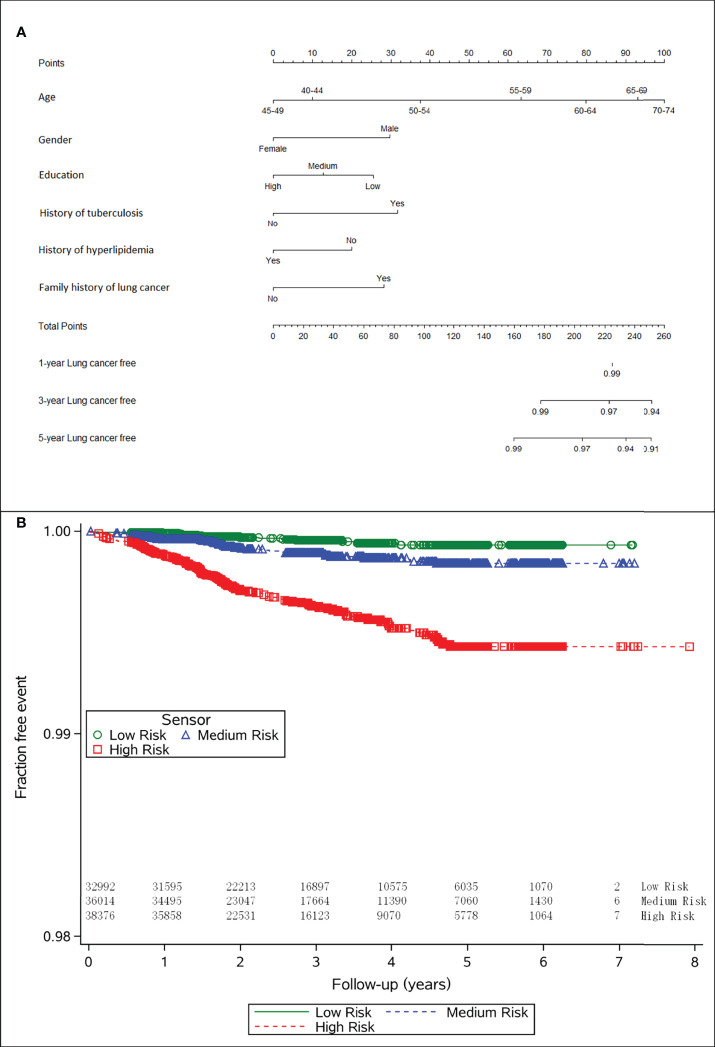
**(A)** Nomogram to calculate the personal 1-, 3- and 5-year risk of lung cancer risk, and **(B)** the lung cancer incidence across different cancer risk categories.

**Table 2** presents the HRs (95% CI) for each predictor. In the training set, age (≥55 years: 3.68,1.60-8.43; ≥60 years: 5.51, 2.48-12.26; ≥65 years: 7.62, 3.43-16.92; ≥70 years: 9.03, 3.79-21.54), gender (male: 2.07, 1.53-2.79), education (low: 1.87, 1.05-3.33; medium: 1.36, 0.81-2.31), family history of lung cancer (2.00, 1.25-3.20), history of tuberculosis (2.16, 0.87-5.37), and history of hyperlipidemia (0.61, 0.40-0.95) were independent risk factors of lung cancer. Thus, we used these variables to build the model. We plotted 1-year, 3-year, and 5-year lung cancer risk prediction nomogram (**Figure 2A**).

**Table 2:** Multivariable Cox-regression prediction model of lung cancer risk in training set.

Variables	*β coefficient*	*se*	HR (95% *CI*)	χ^2^	*P*
Age (years)					
40-44			1.00		
45-49	-0.24	0.52	0.78 (0.28-2.16)	0.22	0.637
50-54	0.67	0.44	1.96 (0.82-4.66)	2.29	0.130
55-59	1.30	0.42	3.68 (1.60-8.43)	9.47	0.002
60-64	1.71	0.41	5.51 (2.48-12.26)	17.53	<0.001
65-69	2.03	0.41	7.62 (3.43-16.92)	24.92	<0.001
70-74	2.20	0.44	9.03 (3.79-21.54)	24.65	<0.001
Gender					
Male	0.73	0.15	2.07 (1.53-2.79)	22.63	<0.001
Female			1.00		
Education^a^					
Low	0.62	0.30	1.87 (1.05-3.33)	4.45	0.035
Medium	0.31	0.27	1.36 (0.81-2.31)	1.33	0.249
High			1.00		
Family history of lung cancer					
No			1.00		
Yes	0.69	0.24	2.00 (1.25-3.20)	8.24	0.004
History of tuberculosis					
No			1.00		
Yes	0.77	0.46	2.16 (0.87-5.37)	2.75	0.097
History of hyperlipidemia					
No			1.00		
Yes	-0.49	0.22	0.61 (0.40-0.95)	4.80	0.028

^a^Low, primary school or below; Medium, junior or senior high school; High, undergraduate or over.

### Predictive Performance of the Model

The risk predictions were stratified into low-, medium-, and high-risk groups and visualized by Kaplan–Meier curves, showing statistically significant differences between the groups by a log-rank test (**Figure 2B**, *P*<0.001).

**Figure 3:**
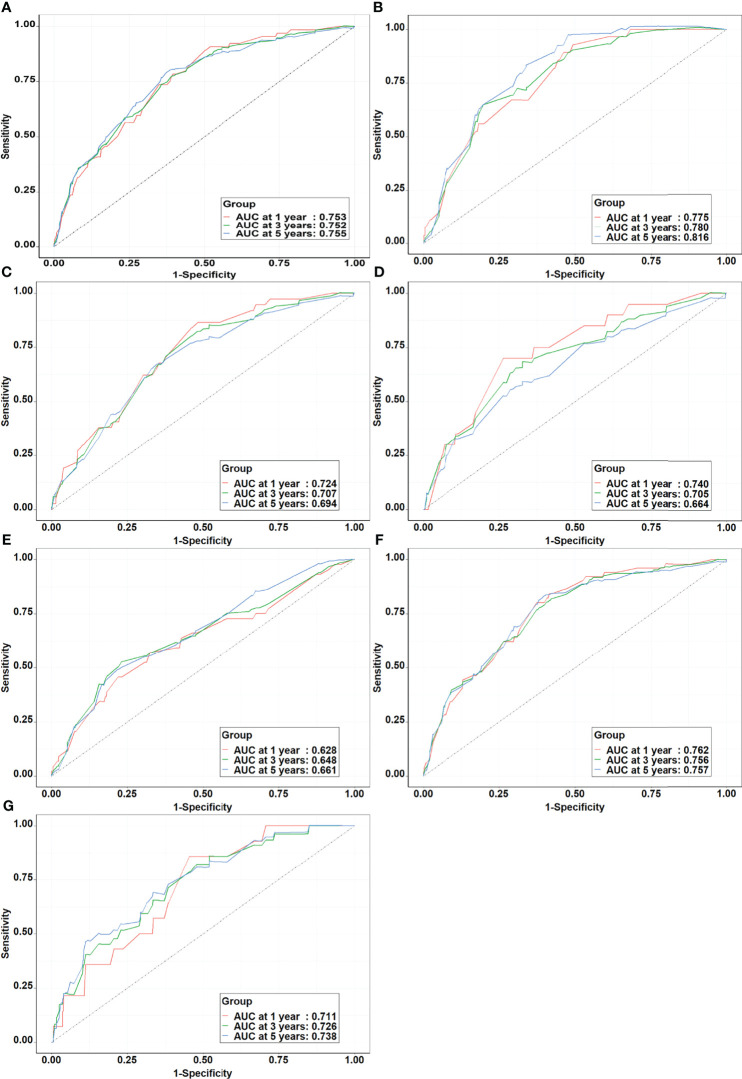
The receiver operating characteristic curves of prediction models in the training set. **(A)** Whole population; **(B)** Male; **(C)** Female; **(D)** Age<60 years; **(E)** Age≥60 years; **(F)** Non-passive smokers; **(G)** Passive smokers.

**Figure 4:**
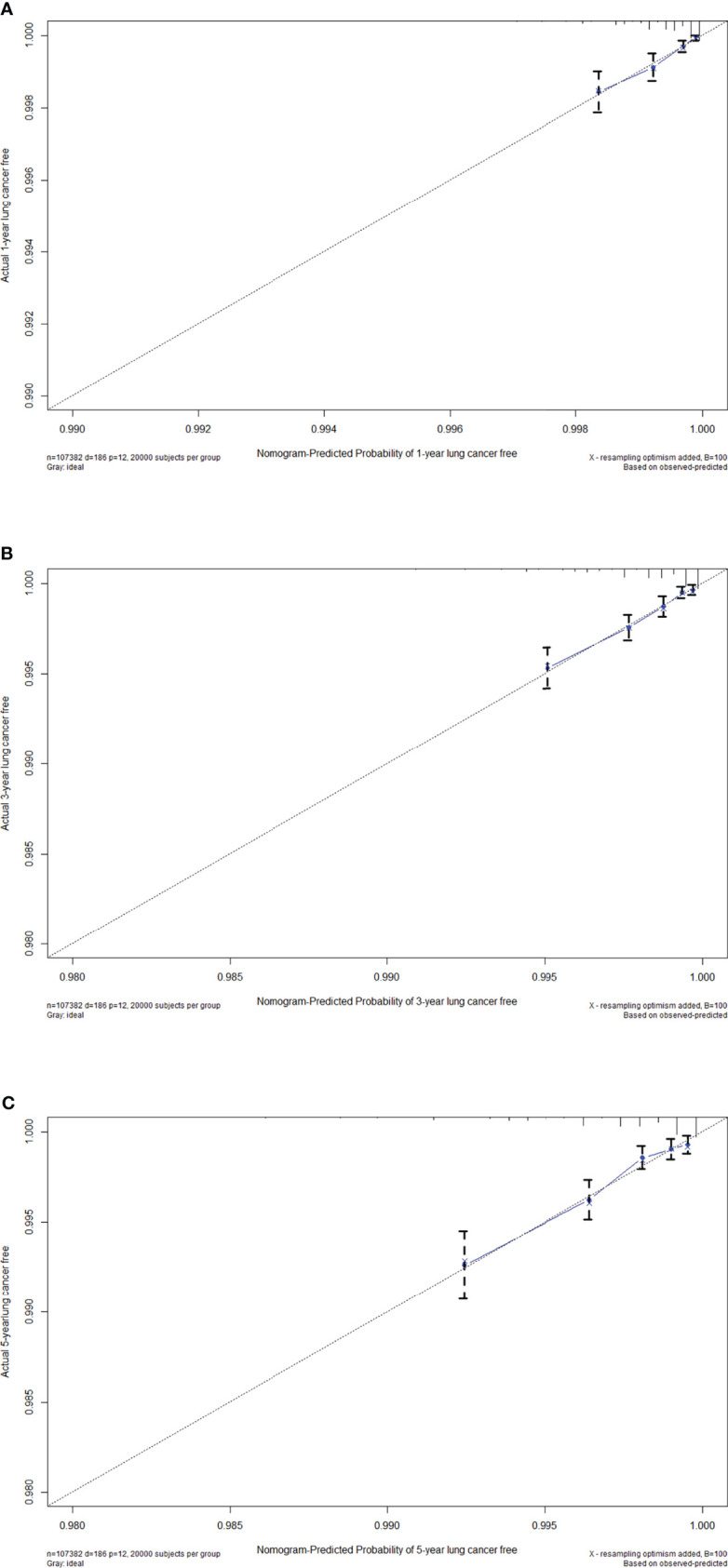
Calibration curves of the nomogram for **(A)** 1-year, **(B)** 3-year and **(C)** 5-year lung cancer free in the training set.

Using this model, the AUC was 0.753, 0.752, and 0.755 for 1-year, 3-year, and 5-year lung cancer risk in the training set, respectively. Stratified analysis by gender showed that the AUC of the model was higher among men (1-year: 0.776, 3-year: 0.780, and 5-year: 0.816) than women (1-year: 0.724, 3-year: 0.707, and 5-year: 0.694). Stratified analysis by age showed that the AUC of the model was higher among younger participants (<60 years) (1-year: 0.740, 3-year: 0.705, and 5-year: 0.664) than elder participants (≥60 years) (1-year: 0.628, 3-year: 0.648, and 5-year: 0.661). When examined by passive smoking status, the model yielded higher AUC for non-passive smokers (1-year: 0.762, 3-year: 0.756, and 5-year: 0.757) than passive smokers (1-year: 0.711, 3-year: 0.726, and 5-year: 0.738) (**Figure 3**). Calibration was satisfactory, with observed risks awfully close to the predicted risks (**Figure 4**).

### Validation of the Lung Cancer Risk Model

The model showed a moderate predictive discrimination in the validation set, with the AUC was 0.668, 0.678, and 0.685 for 1-year, 3-year, and 5-year lung cancer risk (**Supplementary Figure 1**) and the satisfactory calibration of relative risk (**Supplementary Figure 2**).

## Discussion

In this study, using data from a large perspective lung cancer screening cohort studies, we developed and internally validated a simple risk prediction model for lung cancer in non-smokers, based on six widely available variables, including demographics (age, gender, education), comorbidities (tuberculosis, hyperlipidemia) and family history of lung cancer. Our results showed that the model has good discriminatory accuracy and goodness-of-fit for both men and women, non-passive smokers and passive smokers.

For non-smokers, several risk factors for lung cancer have been identified, including passive smoking (47, 48), previous lung diseases [tuberculosis, chronic bronchitis, emphysema, previous lung diseases (COPD)] (49), indoor radon (50), cooking oil fumes (51) and family history of lung cancer (52). The risk factors for lung cancer identified in our study, such as age, gender, family history of lung cancer, history of tuberculosis, are consistent with the findings. The most dominant risk factors for lung cancer in non-smokers is age, and our study showed that elder age was the main risk factor for lung cancer and the risk was more than 9 times higher in age group of 70-74 years than in the age group of 40-44 years. Besides, being male remains a risk factor for lung cancer in non-smokers in our study, even though more than 50% lung cancers were non-smokers in women in Southeast Asia compared to approximately 2–6% in men in Western series (41, 42, 53). Just like other prediction models, such as Bach model (8), LLP (Liverpool Lung Project) model (10) and PLCO_M2012_ model (54), education levels was included in our model as a protection factor.

Another important finding was that history of hyperlipidemia [increased total cholesterol (TC), or triglycerides (TG), or low-density lipoprotein cholesterol (LDL-C), or decreased high-density lipoprotein cholesterol (HDL-C)] exposure might decrease the risk of lung cancer, despite a small effect. Since the 1980s, several epidemiological studies have investigated the associations of TC, TG, and HDL-C with lung cancer risk in non-smokers but have shown markedly contrasting results due to differences in the classification of smoking status, lack of prospective cohort study designs, relatively modest sample sizes and other potential bias (55–58). Lyu etc. (58) conducted a prospective cohort study among over 100 thousand Chinese males and found that both low and high TC levels, both low and high TG levels, and low LDL-C levels increased lung cancer risk in non-smokers. Besides, many studies reported an inverse relationship between TC (56, 59), LDL-C (60) and lung cancer incidence, to some extent, consistent with our findings. More epidemiologic, molecular and biochemical studies are needed to test this hypothesis.

In addition to credible predictors, a risk prediction model should also meet performance standards related to discrimination defined as the ability to distinguish lung cancer cases from controls, and calibration defined as the consistency between observed and predicted risk for lung cancer. The rapid increase in the number of lung cancer risk prediction model studies since 2010 reflects the current need for the use of predictive models to guide population splitting. Initially, models focused on the use of traditional epidemiological risk factors such as age, smoking history, personal history of disease and family history of cancer, such as the Bach model (8), Spitz model (9), LLP model (10) and PLCO_M2012_ model (54). To our knowledge, the present study is one of the few studies to model lung cancer risk prediction among non-smoking men and women in mainland China. It is hard to directly compare the discriminatory performance of risk prediction models as each was developed in different populations with varying baseline risks or lengths of follow-up time. Nevertheless, each of the models’ discriminative ability was relatively similar, with C-statistics ranges from 0.72 to 0.86. Our model showed comparable predictive performance compared with previous studies.

Specific strengths and limitations deserve careful attention when interpreting our results. A major strength of our study is the fact that our analyses were based on a large-scale population-based cancer screening program in mainland of China. Furthermore, the variables included in this model could be easily collected and updated without any imaging, sophisticated testing or calculation. Moreover, the model will not only be used as a practical tool to triage high risk patients in non-smokers, but also have implications for public health measures, such as guidelines for the prevention of lung cancer in non-smokers. However, limitations include that the self-report data might subject to social desirability and recall bias. However, given the good data acquisition and quality control, most information is believed to be reliable. Secondly, the performance of our risk prediction model was not validated on an external dataset. However, the results of the internal validation suggest promisingly that this model will obtain well performance when applied to other populations.

## Conclusions

In summary, we developed and internally validated a simple risk prediction model for lung cancer in non-smokers based on a large-scale lung cancer screening program in China. The model has good discrimination and could be used as a tool for triaging high-risk patients to prevent lung cancer in non-smokers. Further prospective studies are required to validate the model in external populations.

## Data Availability Statement

The datasets for this manuscript are not publicly available because all our data are under regulation of both the National Cancer Center of China and Henan Cancer Hospital. Requests to access the datasets should be directed to Shaokai Zhang, shaokaizhang@126.com.

## Ethics Statement

The studies involving human participants were reviewed and approved by the Ethics Committee of Henan Cancer Hospital (no. 2021-KY-0028-001). The patients/participants provided their written informed consent to participate in this study.

## Author Contributions

Conception and design: L-WG, Z-YL, J-GZ and S-KZ. Statistical Analyses: L-WG and L-YZ. Data acquisition and data interpretation: L-WG, Q-CM, L-YZ, QC, YL, H-FX, R-HK, L-YZ, X-QC, S-ZL, X-BS and S-KZ. Drafting of the article: L-WG. All authors contributed to the article and approved the submitted version.

## Funding

This study was supported by the Natural Science Foundation of Henan Province (No. 212300410261), the Henan Province Medical Science and Technology Tackling Program (SBGJ202001004), the National Key R&D Program of China (2018YFC1315000/2018YFC1315001) and the CAMS Innovation Fund for Medical Science (2019-I2M-2-002).

## Conflict of Interest

The authors declare that the research was conducted in the absence of any commercial or financial relationships that could be construed as a potential conflict of interest.

## Publisher’s Note

All claims expressed in this article are solely those of the authors and do not necessarily represent those of their affiliated organizations, or those of the publisher, the editors and the reviewers. Any product that may be evaluated in this article, or claim that may be made by its manufacturer, is not guaranteed or endorsed by the publisher.
