# Corrigendum: Construction and Validation of a Lung Cancer Risk Prediction Model for Non-Smokers in China

**DOI:** 10.3389/fonc.2022.871848

**Published:** 2022-03-03

**Authors:** Lan-Wei Guo, Zhang-Yan Lyu, Qing-Cheng Meng, Li-Yang Zheng, Qiong Chen, Yin Liu, Hui-Fang Xu, Rui-Hua Kang, Lu-Yao Zhang, Xiao-Qin Cao, Shu-Zheng Liu, Xi-Bin Sun, Jian-Gong Zhang, Shao-Kai Zhang

**Affiliations:** ^1^ Department of Cancer Epidemiology and Prevention, Henan Engineering Research Center of Cancer Prevention and Control, Henan International Joint Laboratory of Cancer Prevention, The Affiliated Cancer Hospital of Zhengzhou University, Henan Cancer Hospital, Zhengzhou, China; ^2^ Department of Cancer Epidemiology and Biostatistics, National Clinical Research Center for Cancer, Key Laboratory of Cancer Prevention and Therapy of Tianjin, Tianjin’s Clinical Research Center for Cancer, Key Laboratory of Molecular Cancer Epidemiology of Tianjin, Key Laboratory of Breast Cancer Prevention and Therapy of the Ministry of Education, Tianjin Medical University Cancer Institute and Hospital, Tianjin, China; ^3^ Department of Radiology, The Affiliated Cancer Hospital of Zhengzhou University, Henan Cancer Hospital, Zhengzhou, China

**Keywords:** lung cancer, risk model, forecasting, validation, non-smokers

In the originally published article, affiliations 1 and 3 were presented incorrectly.

Affiliation 1 was presented as “Department of Cancer Epidemiology and Prevention, Henan Engineering Research Center of Cancer Prevention and Control, Henan International Joint Laboratory of Cancer Prevention, Henan Cancer Hospital, The Affiliated Cancer Hospital of Zhengzhou University, Zhengzhou, China”; it should be “Department of Cancer Epidemiology and Prevention, Henan Engineering Research Center of Cancer Prevention and Control, Henan International Joint Laboratory of Cancer Prevention, The Affiliated Cancer Hospital of Zhengzhou University, Henan Cancer Hospital, Zhengzhou, China”.

Affiliation 3 was presented as “Department of Radiology, Henan Cancer Hospital, The Affiliated Cancer Hospital of Zhengzhou University, Zhengzhou, China”; it should be “Department of Radiology, The Affiliated Cancer Hospital of Zhengzhou University, Henan Cancer Hospital, Zhengzhou, China”.

The authors apologize for these errors and state that this does not change the scientific conclusions of the article in any way. The original article has been updated.

## Publisher’s Note

All claims expressed in this article are solely those of the authors and do not necessarily represent those of their affiliated organizations, or those of the publisher, the editors and the reviewers. Any product that may be evaluated in this article, or claim that may be made by its manufacturer, is not guaranteed or endorsed by the publisher.

