# Supplementary material for: Construction and Validation of a Lung Cancer Risk Prediction Model for Non-Smokers in China
Source: Front Oncol. 2022 Jan 4;11:766939. doi: 10.3389/fonc.2021.766939 (PMC8764453; doi:10.3389/fonc.2021.766939)
Supplement: Supplementary file 1 [file DataSheet_1.docx]

**Supplementary table 1 Baseline characteristics of the study population.**

|  | **Total no. (%)** | **No lung cancer, n (%)** | **Lung cancer, n (%)** | **χ^2^** | ***P-*value** |
| --- | --- | --- | --- | --- | --- |
| All participants | 214764 | 214420 (99.84) | 344 (0.16) |  |  |
| Person-years, median(IQR) | 2.95 (1.71-4.83) | 2.95 (1.71-4.83) | 1.55 (0.83-2.55) |  |  |
| Age, mean±SD, years | 55.19±8.77 | 55.18±8.77 | 60.88±7.53 |  |  |
| Age (years) |  |  |  | 143.23 | <0.001 |
| 40-44 | 27725 (12.91) | 27715 (99.96) | 10 (0.04) |  |  |
| 45-49 | 40218 (18.73) | 40195 (99.94) | 23 (0.06) |  |  |
| 50-54 | 39733 (18.50) | 39694 (99.90) | 39 (0.10) |  |  |
| 55-59 | 31856 (14.83) | 31801 (99.83) | 55 (0.17) |  |  |
| 60-64 | 35433 (16.50) | 35339 (99.73) | 94 (0.27) |  |  |
| 65-69 | 28829 (13.42) | 28742 (99.70) | 87 (0.30) |  |  |
| 70-74 | 10970 (5.11) | 10934 (99.67) | 36 (0.33) |  |  |
| Gender |  |  |  | 21.6 | <0.001 |
| Male | 62930 (29.30) | 62790 (99.78) | 140 (0.22) |  |  |
| Female | 151834 (70.70) | 151630 (99.87) | 204 (0.13) |  |  |
| Race |  |  |  | 0.12 | 0.732 |
| Han nationality | 211132 (98.31) | 210793 (99.84) | 339 (0.16) |  |  |
| Others | 3632 (1.69) | 3627 (99.86) | 5 (0.14) |  |  |
| Education ^a^ |  |  |  | 8.68 | 0.013 |
| Low | 40736 (18.97) | 40655 (99.80) | 81 (0.20) |  |  |
| Medium | 143117 (66.64) | 142888 (99.84) | 229 (0.16) |  |  |
| High | 30911 (14.39) | 30877 (99.89) | 34 (0.11) |  |  |
| BMI (kg/m^2^) |  |  |  | 3.31 | 0.347 |
| <18.5 | 2759 (1.28) | 2753 (99.78) | 6 (0.22) |  |  |
| 18.5-24.0 | 95356 (44.40) | 95192 (99.83) | 164 (0.17) |  |  |
| 24.0-28.0 | 93514 (43.54) | 93369 (99.84) | 145 (0.16) |  |  |
| ≥28.0 | 23135 (10.77) | 23106 (99.87) | 29 (0.13) |  |  |
| Vegetables intake |  |  |  | 0.01 | 0.919 |
| ≥2.5kg/week | 113034 (52.63) | 112852 (99.84) | 182 (0.16) |  |  |
| <2.5kg/week | 101730 (47.37) | 101568 (99.84) | 162 (0.16) |  |  |
| Fruit intake |  |  |  | 0.33 | 0.564 |
| ≥1.25kg/week | 126274 (58.80) | 126077 (99.84) | 197 (0.16) |  |  |
| <1.25kg/week | 88490 (41.20) | 88343 (99.83) | 147 (0.17) |  |  |
| Roughage intake |  |  |  | 1.56 | 0.211 |
| ≥0.5kg/week | 147188 (68.53) | 146963 (99.85) | 225 (0.15) |  |  |
| <0.5kg/week | 67576 (31.47) | 67457 (99.82) | 119 (0.18) |  |  |
| Heavy-slat diet |  |  |  | 0.56 | 0.453 |
| No | 177127 (82.48) | 176838 (99.84) | 289 (0.16) |  |  |
| Yes | 37637 (17.52) | 37582 (99.85) | 55 (0.15) |  |  |
| Heavy-grease diet |  |  |  | 0.24 | 0.624 |
| No | 179410 (83.54) | 179126 (99.84) | 284 (0.16) |  |  |
| Yes | 35354 (16.46) | 35294 (99.83) | 60 (0.17) |  |  |
| Cooking oil fume exposure |  |  |  | 3.05 | 0.081 |
| None or a little | 190226 (88.57) | 189911 (99.83) | 315 (0.17) |  |  |
| A lot | 24538 (11.43) | 24509 (99.88) | 29 (0.12) |  |  |
| Passive smoking |  |  |  | 6.64 | 0.010 |
| No | 153216 (71.34) | 152949 (99.83) | 267 (0.17) |  |  |
| Yes | 61548 (28.66) | 61471 (99.87) | 77 (0.13) |  |  |
| Alcohol Drinking |  |  |  | 1.07 | 0.586 |
| Never | 192208 (89.50) | 191895 (99.84) | 313 (0.16) |  |  |
| Current | 18994 (8.84) | 18969 (99.87) | 25 (0.13) |  |  |
| Former | 3562 (1.66) | 3556 (99.83) | 6 (0.17) |  |  |
| Physical activity |  |  |  | 13.86 | <0.001 |
| Moderate or no | 112664 (52.46) | 112518 (99.87) | 146 (0.13) |  |  |
| Heavy | 102100 (47.54) | 101902 (99.81) | 198 (0.19) |  |  |
| Family history of lung cancer |  |  |  | 4.57 | 0.033 |
| No | 199304 (92.80) | 198995 (99.84) | 309 (0.16) |  |  |
| Yes | 15460 (7.20) | 15425 (99.77) | 35 (0.23) |  |  |
| History of chronic respiratory disease |  |  |  | 2.16 | 0.141 |
| No | 186205 (86.70) | 185916 (99.84) | 289 (0.16) |  |  |
| Yes | 28559 (13.30) | 28504 (99.81) | 55 (0.19) |  |  |
| History of tuberculosis |  |  |  | 2.72 | 0.099 |
| No | 212389 (98.89) | 212052 (99.84) | 337 (0.16) |  |  |
| Yes | 2375 (1.11) | 2368 (99.71) | 7 (0.29) |  |  |
| History of chronic bronchitis |  |  |  | 0.79 | 0.375 |
| No | 192898 (89.82) | 192594 (99.84) | 304 (0.16) |  |  |
| Yes | 21866 (10.18) | 21826 (99.82) | 40 (0.18) |  |  |
| History of emphysema |  |  |  | 2.08 | 0.149 |
| No | 213105 (99.23) | 212766 (99.84) | 339 (0.16) |  |  |
| Yes | 1659 (0.77) | 1654 (99.70) | 5 (0.30) |  |  |
| History of asthma bronchiectasis |  |  |  | 0.15 | 0.699 |
| No | 209049 (97.34) | 208713 (99.84) | 336 (0.16) |  |  |
| Yes | 5715 (2.66) | 5707 (99.86) | 8 (0.14) |  |  |
| History of hyperlipidemia |  |  |  | 0.28 | 0.598 |
| No | 183211 (85.31) | 182921 (99.84) | 290 (0.16) |  |  |
| Yes | 31553 (14.69) | 31499 (99.83) | 54 (0.17) |  |  |

a. Low, primary school or below; Medium, junior or senior high school; High, undergraduate or over.

Abbreviations: IQR, Interquartile range; BMI, body mass index.

**
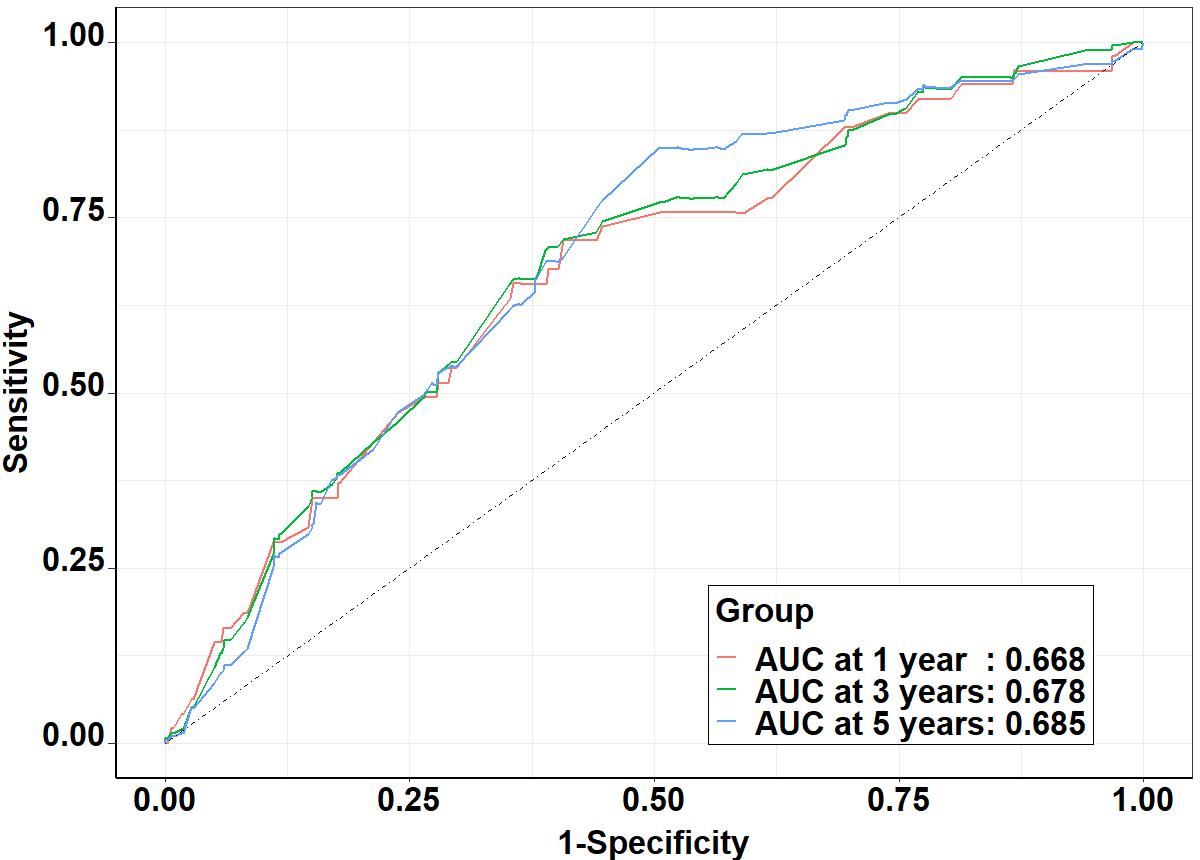
**

**Supplementary Figure 1. The receiver operating characteristic curves of prediction models in the validation set.**


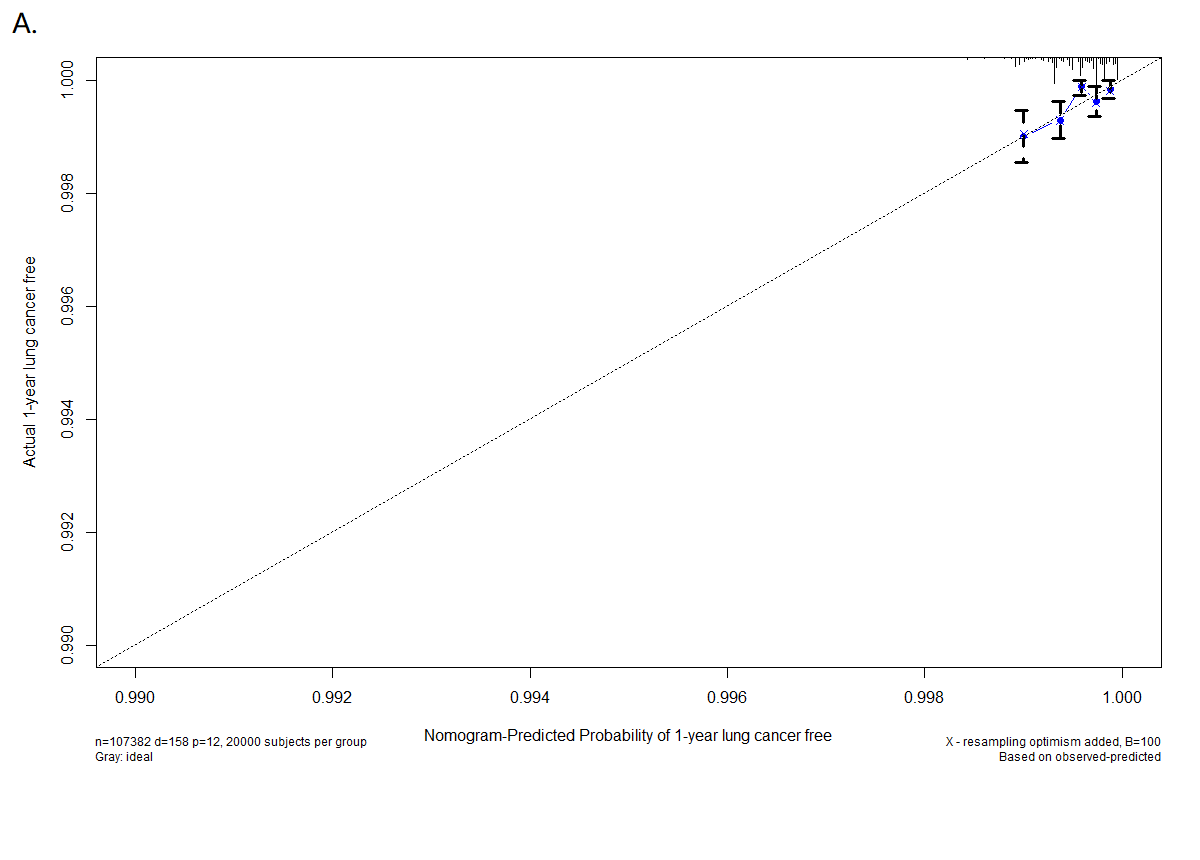


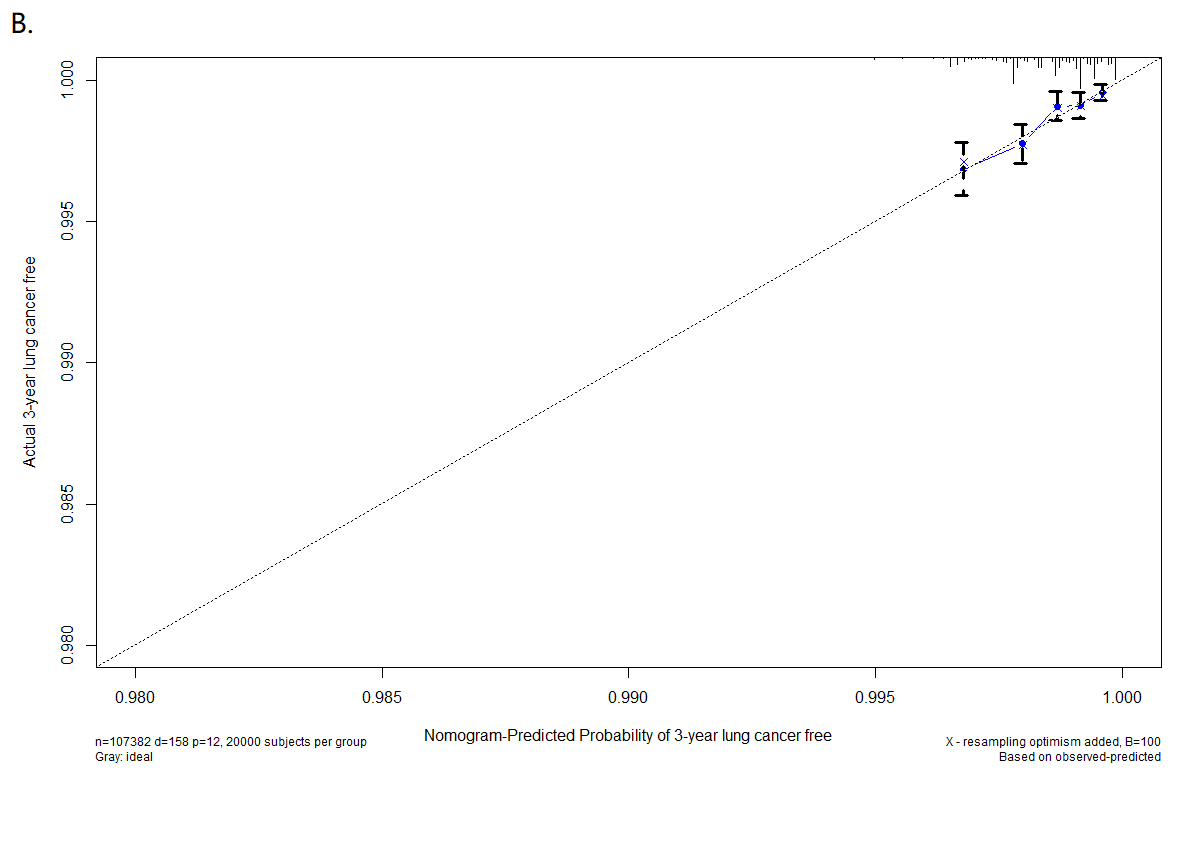


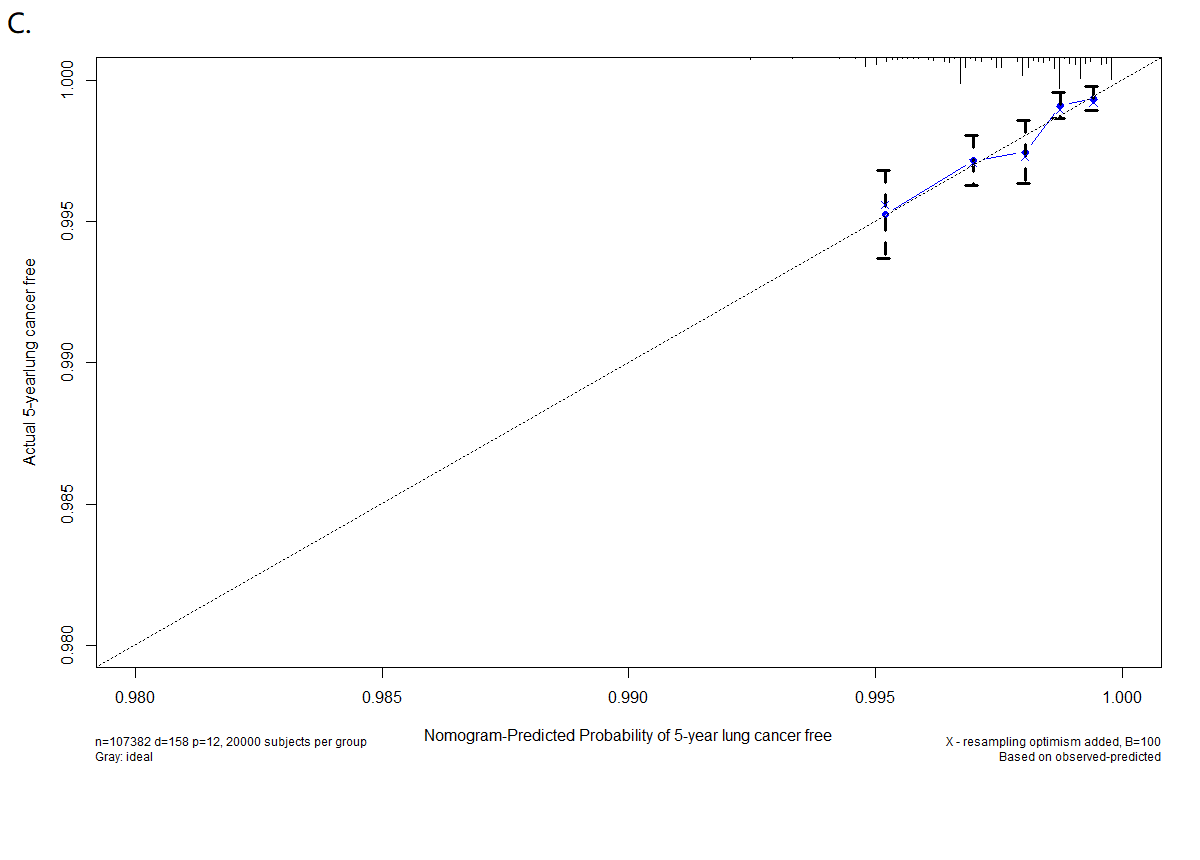


**Supplementary Figure 2. Calibration curves of the nomogram for (A) 1-year, (B) 3-year and (C) 5-year lung cancer free in the validation set.**
